# Proximal Ultrasound-Guided Posterior Tibial Nerve Block for the Removal of Calcaneal Hardware

**DOI:** 10.7759/cureus.41047

**Published:** 2023-06-27

**Authors:** Vicente Garcia Tomas, Alexander M DeLeon, Paul A Johnson, Keziah Vargas, Sarah MacLyman, Brian Chung

**Affiliations:** 1 Anesthesiology, Northwestern University Feinberg School of Medicine, Chicago, USA

**Keywords:** local anesthesia, proximal posterior tibial block, ultrasound guidance, hardware removal, ankle block

## Abstract

The anesthetic technique for calcaneal surgery has been reported to include peripheral nerve blocks, such as a sciatic block in the popliteal fossa, followed by intraoperative sedation. Sciatic nerve blocks are associated with limb weakness and fall risk. We present a case of a patient presenting for outpatient calcaneal surgery. The anesthetic plan consisted of a proximal, ultrasound-guided, single-injection selective posterior tibial nerve block followed by intraoperative sedation. The nerve block was performed, surgery concluded, and the patient received six hours of postoperative analgesia. Once the nerve block effects receded, the postoperative pain was managed with only over-the-counter analgesics while the patient was at home. We recommend an ultrasound-guided proximal posterior tibial nerve block for outpatient surgery involving the calcaneus to preserve lower extremity motor strength and provide postoperative analgesia.

## Introduction

Peripheral nerve blocks have been described as relieving pain associated with calcaneal surgery [1,2]. The preferred peripheral nerve block site for calcaneal surgery is the sciatic nerve block in the popliteal fossa [1]. Unfortunately, lower extremity blocks are associated with falls and the potential for nerve injuries [3-6]. A proximal land-mark-based approach to the posterior tibial nerve was described yet did not demonstrate an improved onset or success rate compared to a distal nerve stimulator approach [7]. Calcaneal branches, including the inferior, medial, and lateral calcaneal nerves, are distal branches of the posterior tibial nerve and provide sensation to the calcaneus. The calcaneal nerves have variable branch points, which could be proximal to the needle entry point of a distal posterior nerve block [8,9]. A proximal approach to the posterior nerve block would be ideal for calcaneal surgery by avoiding the lower extremity weakness associated with popliteal sciatic nerve blockade while maintaining the benefits of postoperative analgesia and early post-anesthesia recovery room discharge.

We present a novel approach to surgical anesthesia and postoperative analgesia utilizing a proximal ultrasound-guided posterior tibial nerve block for outpatient calcaneal surgery.

## Case presentation

**Figure 1:**
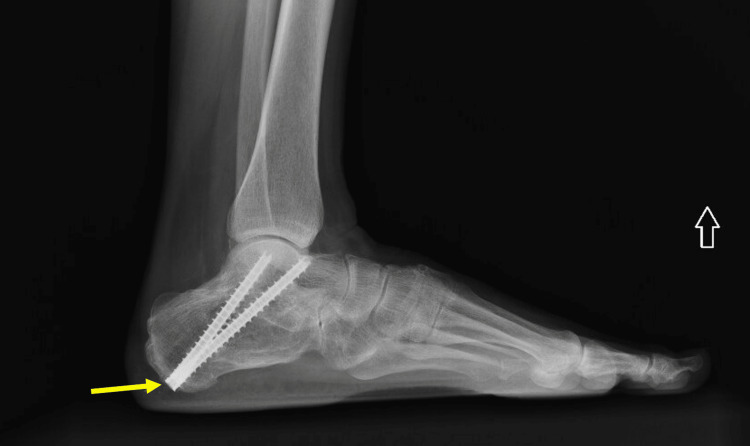
Left Ankle Lateral Plain Film Radiograph The planned surgery was the removal of the two screws (yellow arrow) in the left calcaneus.

A 58-year-old, 94-kilogram woman with a body mass index of 32.63 kg/m2 presented for hardware removal from her left calcaneus (Figure 1). Her medical history was significant for essential hypertension, moderate obstructive sleep apnea, and obesity. Eight years before surgery, the patient suffered a fall from a ladder and fractured her left calcaneus. She continued to have pain after the open reduction and internal fixation of the calcaneal fracture, and thus, four years later, she underwent a subtalar fusion. She was undergoing medical treatment for her chronic calcaneal pain, which marginally improved following subtalar fusion. Before this scheduled surgery, she complained of symptomatic hardware irritation and requested the removal of her calcaneal hardware. Her medications included amlodipine, atenolol, gabapentin, and duloxetine.

The anesthesia team initially planned to perform an ultrasound-guided popliteal fossa sciatic block followed by intraoperative sedation. The block would commit the patient to use a pair of crutches after the surgery for 12 to 24 hours. Thus, the anesthesia team changed the plan to a long-acting ultrasound-guided, proximal posterior tibial nerve block with intraoperative sedation.

**Figure 2:**
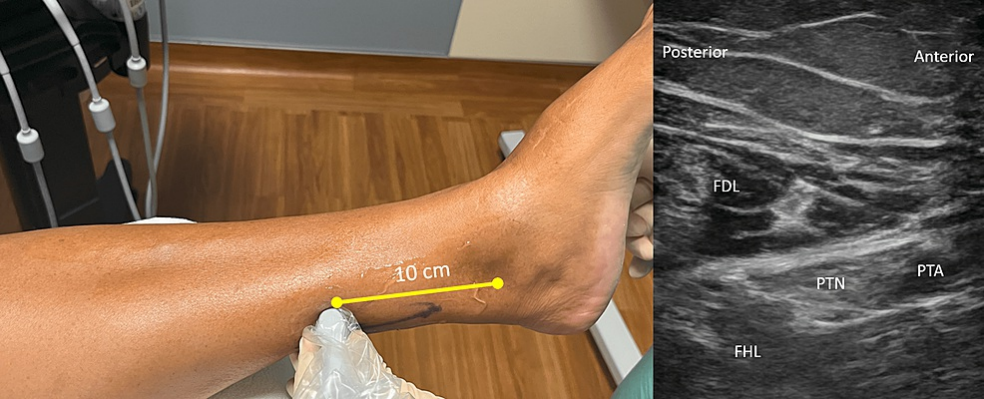
Placement of Ultrasound Probe and Labeled Sonoanatomy The ultrasound was placed 10 cm from the left medial malleolus. The sonoanatomy is labeled. FDL = Flexor digitorum longus, PTN = Posterior tibial nerve, PTA = Posterior tibial artery, FHL = Flexor hallucis longus, and cm = Centimeters.

The patient was monitored throughout the sedation and peripheral nerve block procedure, consistent with the American Society of Anesthesiologists recommendations [10]. The patient received premedication for the nerve block, which included 2 milligrams of midazolam and 100 micrograms of fentanyl. Under ultrasound guidance (Sonosite PX, 19-5 linear high-frequency probe; Bothell, WA, USA), the posterior tibial nerve was visualized 10 centimeters proximal to the medial malleolus (Figure 2). Under direct visualization, 10 milliliters of 0.5% bupivacaine was deposited perineurally using a 22-gauge, 50-millimeter hyperechoic nerve block needle (SonoPlex II Facet S, Pajunk, Alpharetta, GA, USA). The puncture site for the block needle was posterior-medial (i.e., near the Achilles tendon), and the needle was visualized in-plane. The patient demonstrated anesthesia in the calcaneal region within 30 minutes, and strength to plantar flexion was 5 out of 5.

The patient was transported to the operating room, and the surgical incision occurred 39 minutes after nerve block completion. Intraoperative sedation consisted of a propofol infusion at 75 micrograms per kilogram per minute. The surgical duration was 27 minutes, and the propofol was discontinued. The patient was transferred to phase two recovery, discharged from the ambulatory surgicenter one hour later, and required no analgesics while in the recovery room. Her visual analog pain scale (VAS) score was 0 out of 10 on discharge. At home, the patient's activity was weight-bearing as tolerated, and she used a single crutch. She stated on follow-up that she only required the crutch for slight instability from the loss of sensation in her foot, yet she was able to ambulate without the crutch three hours later. After the sensation in her foot returned, she rated her VAS score as 2-3 out of 10, easily controlled with acetaminophen and ibuprofen.

On one month follow-up, the patient reported no complications associated with the nerve block.

## Discussion

Regional anesthesia is widely used throughout the United States for its favorable side effect profile, postoperative analgesia, early discharge, and reduction in unexpected hospital admissions [11]. Complications related to regional anesthesia are rare, yet for lower extremity blockade, fall risk continues to be a problem [3-6]. The proximal blockade of the posterior tibial nerve was evaluated by Doty et al. [7]. Their group showed no benefit compared to a distal approach, yet the proximal block was not assessed for surgery involving the calcaneus [7]. Avoiding the sciatic nerve and, thus, calf weakness for patients undergoing calcaneal surgery while providing the benefits of postoperative pain relief and early discharge would be an ideal anesthetic option for calcaneal surgery.

The decision to attempt a proximal approach to the posterior tibial nerve was based on the goal of preserving the gastrocnemius and soleus muscle function, thus reducing the risk of falls [6]. Using ultrasound, the posterior tibial nerve was traced from inferior to the medial malleolus to a position 10 centimeters from the malleolus (Figure 2). The decision for the location to perform the nerve block was focussed on balancing the risks of being distal to the calcaneal branch point with the chances of causing a motor block in the flexors of the foot (flexor hallucis longus and flexor digitorum longus).

The duration of analgesia was six hours post-surgery. The popliteal sciatic block using 0.5% bupivacaine often provides analgesia for more than 20 hours [12]. Despite the shorter duration of analgesia, the patient was discharged from the recovery room pain-free and successfully managed her postoperative pain with over-the-counter medications. The authors of this report argue that the shorter duration of action is worth the tradeoff of maintaining calf muscle function and thus mitigating fall risk. The patient received no postoperative or intraoperative opioids and therefore experienced no adverse opioid-induced effects, which would have delayed discharge.

The limitations of this report are similar to all case reports in that it is based on a single patient. The generalizability with small sample sizes is thus weak, and this report is intended to serve as a backbone for future research.

## Conclusions

Outpatient orthopedic surgery continues to benefit from regional anesthetic techniques due to the analgesic benefits and evidence of early discharge from the post-anesthesia recovery unit. Side effects of lower extremity peripheral nerve blocks, such as leg weakness, place a patient at increased risk for falls. Proximal selective blockade of the posterior tibial nerve provides surgical anesthesia while preserving most of the motor function of the leg and is a valuable option for outpatient calcaneal surgery.
